# Beam-induced damage on diffractive hard X-ray optics

**DOI:** 10.1107/S0909049510028487

**Published:** 2010-09-02

**Authors:** K. Nygård, S. Gorelick, J. Vila-Comamala, E. Färm, A. Bergamaschi, A. Cervellino, F. Gozzo, B. D. Patterson, M. Ritala, C. David

**Affiliations:** a Paul Scherrer Institut, CH-5232 Villigen PSI, Switzerland; b Department of Chemistry, University of Helsinki, FI-00014 Helsinki, Finland

**Keywords:** diffractive X-ray optics, radiation damage

## Abstract

The issue of beam-induced damage on diffractive hard X-ray optics is addressed. For this purpose a systematic study on the radiation damage induced by a high-power X-ray beam is carried out in both ambient and inert atmospheres. Diffraction gratings fabricated by three different techniques are considered: electroplated Au gratings both with and without the polymer mold, and Ir-coated Si gratings. The beam-induced damage is monitored by X-ray diffraction and evaluated using scanning electron microscopy.

## Abstract 2

Beam-induced damage on diffractive hard X-ray optics is studied by means of X-ray diffraction and scanning electron microscopy.

## 1. Introduction

Many modern synchrotron radiation techniques, such as X-ray microscopy^1^ and coherent diffractive imaging (Marchesini *et al.*, 2003 ▶), rely on an accurate control of the X-ray wavefront. In the hard X-ray regime up to 12 keV photon energies, this can be conveniently done using diffractive optics; depending on the spatial arrangement of the diffractive optical device, the interference pattern results, for example, in a sub-50 nm-sized bright spot in the focus of a Fresnel zone plate (Yin *et al.*, 2006 ▶; Chu *et al.*, 2008 ▶; Chen *et al.*, 2008 ▶) or a rectangular flat-top illumination for a beam-shaping condenser (Jefimovs *et al.*, 2008 ▶).

Since diffractive X-ray optics typically consist of nano­fabricated structures on thin support membranes they are prone to beam-induced damage. In fact, the radiation damage induced by the intense X-ray beams available at modern insertion-device beamlines is already a limiting factor concerning the lifetime of diffractive X-ray optics. This is expected to be an even more pronounced problem at the future X-ray free-electron lasers (Vartanyants *et al.*, 2007 ▶). Despite the importance of this issue, no systematic study of beam-induced damage on diffractive X-ray optics has to our knowledge been carried out.

In terms of possible beam-induced damage mechanisms we expect the diffractive X-ray optical devices to be sensitive to, for example, the following effects:

(i) Hydrocarbon chain bond breaking (scissioning) in polymers and subsequent mass loss.

(ii) Destruction by chemical reactions with ozone or oxygen radicals formed in the surrounding atmosphere.

(iii) Breaking of interfaces, crack formation and/or melting owing to the radiation heat load and the subsequent increase in temperature.

Given the complexity of these mechanisms, an experimental approach is necessary to address the issue of beam-induced damage on diffractive X-ray optics.

In this research paper we report the first quantitative study of beam-induced damage on diffractive X-ray optics. For this purpose we subjected different types of diffraction gratings to a high-power X-ray beam in both ambient and inert atmospheres. We evaluated the beam-induced damage using both X-ray diffraction (XRD), *i.e.* by monitoring the diffraction efficiency of the grating as a function of irradiation, and scanning electron microscopy (SEM). We expect the results of the present study to guide the planning and fabrication of future diffractive X-ray optics.

## 2. Materials and methods

Throughout this study we used three different types of linear diffraction gratings as diffractive optical elements:

(i) Pi/Au: gratings consisting of alternating polyimide and Au lines were fabricated in a two-step approach. First, a polyimide mold was manufactured by means of electron-beam lithography and dry etching. In order to increase the mechanical stability of the polymer mold, buttresses were added between the polymer lines. Second, the polyimide mold was filled with Au by means of electroplating. Details of the grating fabrication can be found elsewhere (Jefimovs *et al.*, 2007 ▶).

(ii) Au: as for the pi/Au gratings except that the polymer mold was removed. Consequently, the gratings consist of segmented Au lines. The reader is referred to Gorelick *et al.* (2010*a* ▶,*b* ▶) for details about the grating fabrication.

(iii) Ir/Si: diffraction gratings etched into Si and coated with a 55 nm-thick Ir layer using atomic layer deposition. Details of the grating fabrication have been published elsewhere (Vila-Comamala *et al.*, 2009 ▶).

**Figure 1:**
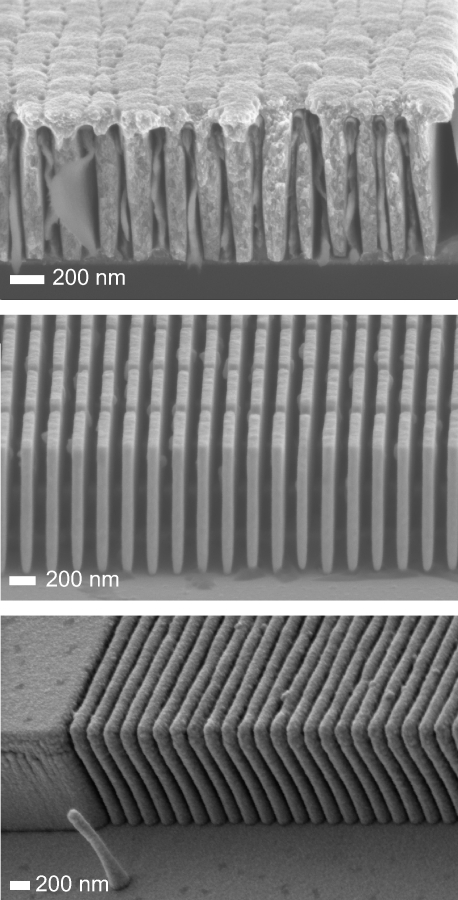
SEM images of the different types of gratings used in this study: overplated pi/Au (top), segmented Au (middle) and Ir/Si gratings (bottom).

Unfortunately the present experimental set-up did not allow us to study Si-based high-heat-load diffractive optics (Vila-Comamala *et al.*, 2008 ▶), owing to a relatively low diffraction efficiency of these gratings. All of the gratings had a period of 100–200 nm, a depth of approximately 1 µm, a duty cycle of ∼0.5 (*i.e.* equal width of lines and spaces), and a size of 200 µm × 200 µm. Throughout this study we used 30–35 µm-thick Si support membranes. SEM images of the different types of gratings are presented in Fig. 1 ▶.

The experiment was carried out at the Material Science beamline of the Swiss Light Source (Patterson *et al.*, 2005 ▶). In order to maximize the incident flux on the sample we did not use a monochromator. Since the mirror suppresses the high-energy part and the absorption in the windows and in the air path the low-energy part of the broad-band radiation emitted from the wiggler source, the resulting ‘pink’ X-ray beam had an effective X-ray energy *E* ≃ 10.3 keV (wavelength λ ≃ 1.2 Å), as determined from the positions of the diffraction peaks, and a moderate energy resolution Δ*E*/*E* ≃ 0.15, as determined from simulations. The incident X-ray beam had a size of approximately 1.0 mm × 0.1 mm (horizontal × vertical) at the sample position and it was focused onto the detector plane in order to maximize the angular resolution. The samples were inserted into a chamber allowing exposures in both ambient atmosphere and in vacuum, and the diffracted X-rays were collected in transmission geometry 1.5 m downstream of the sample using the MYTHEN microstrip detector (Bergamaschi *et al.*, 2009 ▶).

Because of beam-hardening effects,^2^ we carried out the experiment in cycles of the following two steps. First, we exposed the grating with a high-power X-ray beam using the minimum wiggler gap. The sample was exposed with an average flux density *I*_0_ ≃ 6 × 10^14^ photons s^−1^ mm^−2^, as estimated using a power meter (Coherent Inc.). Next, we carried out an XRD experiment with a less intense X-ray beam using the maximum wiggler gap. Owing to the changing of the wiggler gap and the attenuator settings in order to collect XRD data, we had dead-times of approximately 7 min between subsequent exposures with the high-power X-ray beam. Effectively this corresponds to cooling of the sample in between exposures. Finally, we also exposed a set of pi/Au gratings to different amounts of dose in order to perform a systematic SEM inspection of the gratings as a function of irradiation.

Some implications of the present experimental set-up should be noted. (i) The beam profile across the grating is uneven. Consequently, different parts of the grating are irradiated with different dose, an effect which influences the quantitative numbers derived from the experiment. Since the beam profile is kept constant throughout the experiment, this effect cancels out when comparing the results for different gratings or different atmospheres. (ii) Throughout this study, we quantify the beam-induced damage in terms of the integrated average flux density, *N*_0_. Since the spectral flux of the high-power X-ray beam is not changed during the experiment, the absorbed dose is proportional to *N*_0_. Assuming a thin sample and the effective X-ray energy *E*, the absorbed dose is given by 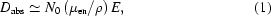
where (μ_en_/ρ) denotes the mass energy-absorption coefficient (Seltzer, 1993 ▶). This gives an average absorbed dose *D*_abs_ ≃ 1.7*N*_0_(μ_en_/ρ) × 10^−10^ Gy, where *N*_0_ and (μ_en_/ρ) are given in units of photons mm^−2^ and cm^2^ g^−1^, respectively. At 10 keV, the tabulated mass energy-absorption coefficients for polyimide, Si, Ir and Au are (μ_en_/ρ)_pi_ ≃ 2.9, (μ_en_/ρ)_Si_ ≃ 33, (μ_en_/ρ)_Ir_ ≃ 100 and (μ_en_/ρ)_Au_ ≃ 110 cm^2^ g^−1^, respectively.

## 3. Results and discussion

In order to quantify the effect of beam-induced damage on the normalized diffraction efficiency η, we introduce an *ad hoc* Beer–Lambert-type model, 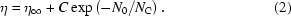
Here 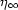 denotes the asymptotic limit of the normalized diffraction efficiency and *C* denotes a constant. The critical integrated flux density, *N*_C_, describes the radiation dose at which 63%, or more specifically the fraction (1 − e^−1^), of the total radiation damage has occurred. We note that equation (2) is formally equivalent to models previously used to quantify beam-induced damage in polymers (Coffey *et al.*, 2002 ▶; Beetz & Jacobsen, 2003 ▶). In the present study, however, the functional form of η may in part be influenced by the uneven beam profile. Nonetheless, 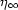 and *N*_C_ can be used as metrics to compare beam-induced damage in different atmospheres or on different types of gratings.

**Figure 2:**
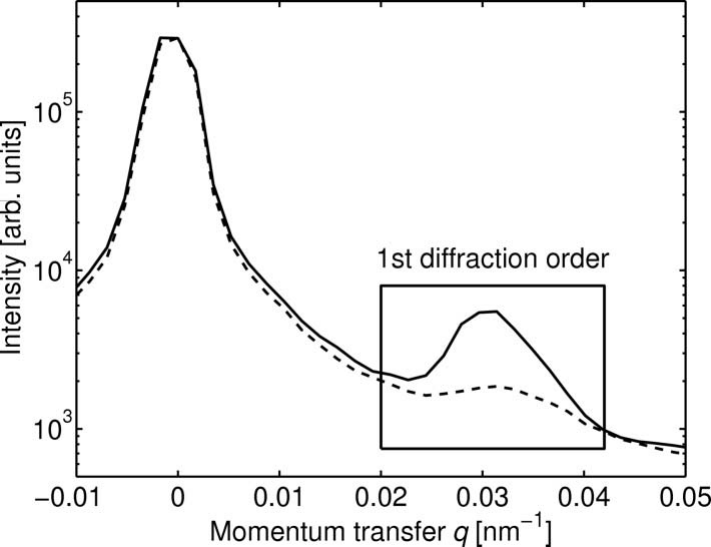
The diffraction pattern obtained before (solid line) and after (dashed line) exposing a pi/Au grating to approximately 11 × 10^17^ photons mm^−2^ using a high-power X-ray beam. Owing to intrinsic symmetry of the diffraction pattern, only positive momentum transfers are shown.

To visualize the effects of beam-induced damage on diffractive hard X-ray optics, we consider a case study of  pi/Au gratings in air. In Fig. 2 ▶ we present typical diffraction patterns obtained before and after exposing a grating to approximately 11 × 10^17^ photons mm^−2^ using the high-power X-ray beam. The effect of the exposure on the diffraction efficiency of the grating is readily observed.

**Figure 3:**
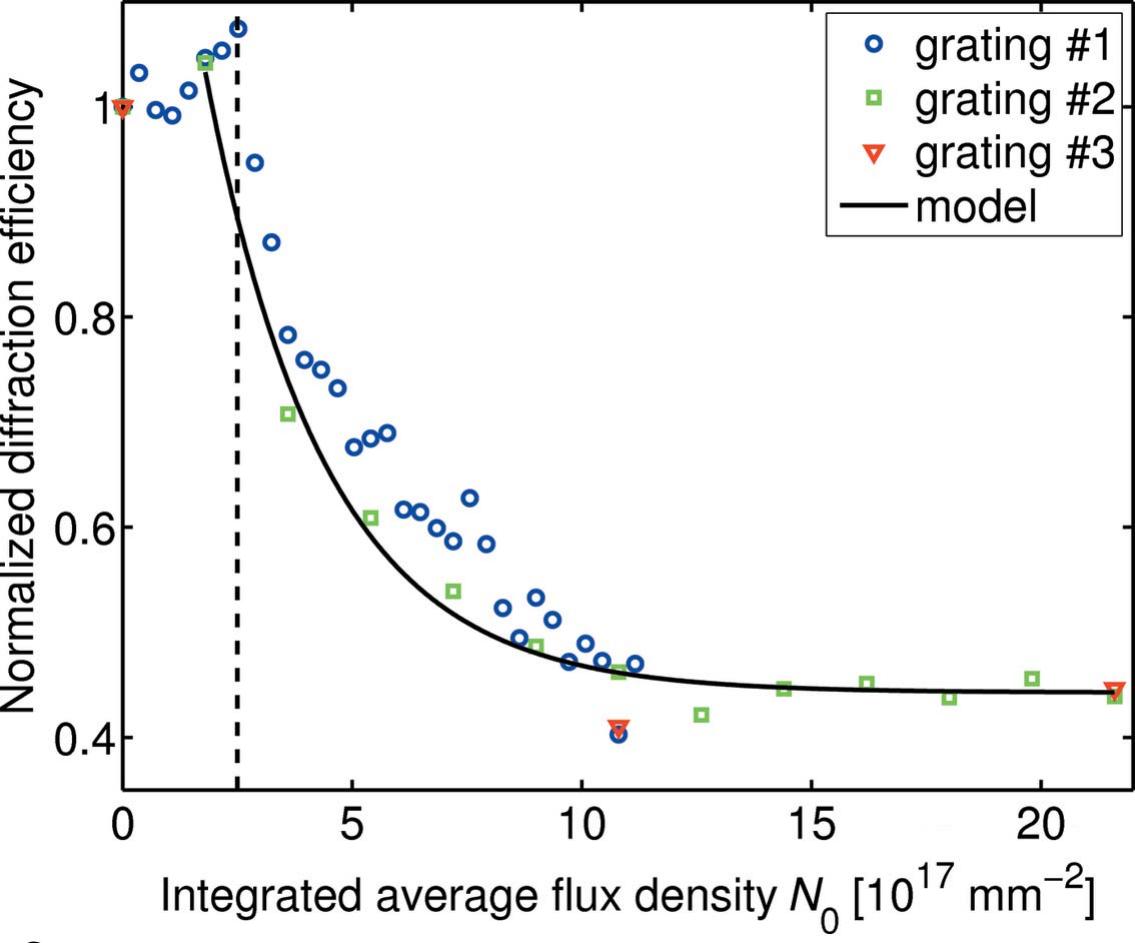
Normalized diffraction efficiency as a function of irradiation for three different pi/Au gratings in air. The solid line depicts a fit of equation (2) to the data of grating #2. The dashed vertical line denotes the threshold value *N*_th_.

From the diffraction pattern we can determine the diffraction efficiency of the grating by dividing the diffracted intensities of the first and zeroth diffraction orders. In order to facilitate comparison between different gratings, we further normalize the diffraction efficiencies for each grating with the value obtained prior to exposure with the high-power X-ray beam (*i.e.* for *N*_0_ = 0). In Fig. 3 ▶ we present the normalized diffraction efficiency as a function of irradiation for three different pi/Au gratings in air. We observe a characteristic decay of the diffraction efficiency with increasing dose. From the data of Fig. 3 ▶ we draw the following conclusions. (i) The radiation damage does not depend on the frequency of the XRD measurements. Although we have no direct measurement of the sample temperature during the experiment, this observation implies that the temperature increase induced by irradiation is not the primary cause of the observed radiation damage. (ii) The diffraction efficiency is unaffected by the irradiation up to a threshold of *N*_th_ ≃ (2.5 ± 0.2) × 10^17^ photons mm^−2^. We have verified this observation for four different pi/Au gratings in air. However, we did not collect XRD patterns with a sufficient frequency from the Au gratings to determine whether this threshold is specific to polymer-containing gratings. (iii) For doses exceeding *N*_th_, we observe an exponential decay of the diffracted intensity. For comparison, we also show a fit of equation (2) to the decaying part of the data obtained for one of the gratings (grating #2). For pi/Au gratings in air, we obtain a critical fluence of *N*_C_ ≃ (3.0 ± 0.5) × 10^17^ photons mm^−2^. (iv) Typically, the asymptotic limit of the normalized diffraction efficiency is non-vanishing, *i.e.* 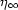 ≠ 0. Moreover, we also observe a scatter in the values of 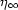, which is of order 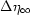 ≃ 0.1. A possible explanation for these findings is the following. When electroplating the polymer molds, some of the gratings were overplated (*i.e.* covered) with Au (see Fig. 1 ▶). The overplated Au effectively encapsulates the polymer matrix, thereby blocking the access of atmospheric gases to the polymer. This, in turn, increases the chemical inertness of the gratings, leading to larger values of 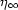.

**Figure 4:**
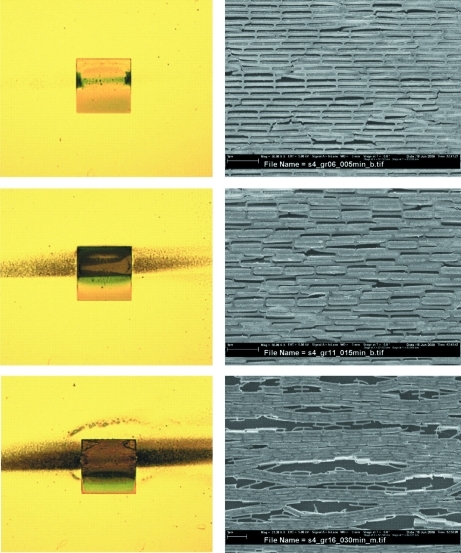
Microscope images of pi/Au diffraction gratings that were exposed using a high-power X-ray beam with approximately 0.6 × 10^17^ (top), 1.8 × 10^17^ (middle) and 3.6 × 10^17^ photons mm^−2^ (bottom) of effective energy *E* ≃ 10.3 keV. Left: optical microscope images. The 200 µm × 200 µm square is the grating, while the dark horizontal stripe is induced by the high-power X-ray beam. Right: high-magnification SEM images of selected regions of the gratings.

In order to gain more insight into beam-induced damage on pi/Au gratings in air, we have also carried out a series of optical microscopy and SEM inspections. These are presented in Fig. 4 ▶, for exposures with *N*_0_ = 0.6 × 10^17^, 1.8 × 10^17^ and 3.6 × 10^17^ photons mm^−2^. As a general rule, we observe an increasing amount of defects with increasing dose. For the largest dose shown in Fig. 4 ▶ we observe regions where the periodic structure is completely destroyed. This implies that the dominant radiation-damage effect is the breaking of the interface between Au and the plating base. However, locally the sample still consists of regions with intact periodic structures, an effect which explains the non-vanishing value of 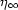.

**Figure 5:**
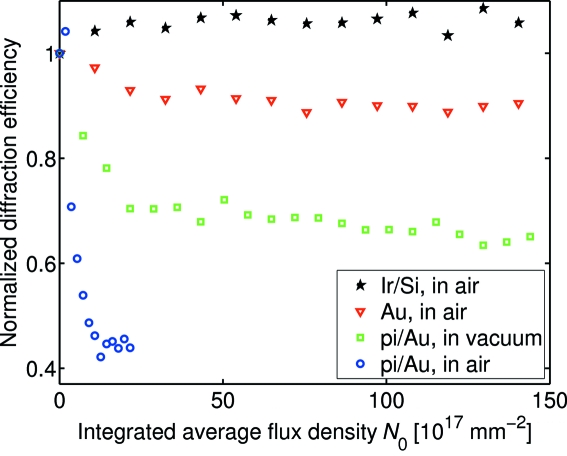
Normalized diffraction efficiencies as a function of irradiation of pi/Au gratings in air (circles), pi/Au gratings in vacuum (squares), Au gratings in air (triangles) and Ir/Si gratings in air (stars).

**Figure 6:**
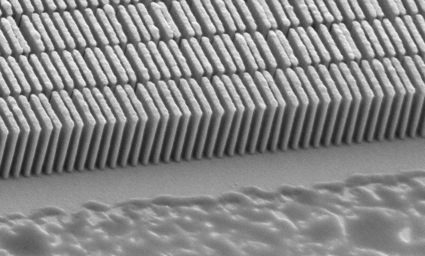
SEM image of the Au grating (triangles in Fig. 5 ▶) after irradiation.

Next, we consider the effect of different atmospheres and different types of gratings. This is presented in Fig. 5 ▶ as the normalized diffraction efficiency for pi/Au gratings in air and in vacuum as well as Au and Ir/Si gratings in air. From the data we make the following observations. (i) The use of a vacuum environment significantly improves the resistance of pi/Au gratings to X-rays, yielding the values 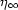 ≃ 0.7 and *N*_C_ ≃ 13 × 10^17^ photons mm^−2^. This observation conforms with previous studies of radiation damage in polymers, in which the use of an inert He atmosphere was found to slow down the radiation damage (Coffey *et al.*, 2002 ▶). (ii) The removal of the polymer mold strongly increases the resistance to X-rays. For the Au gratings in air, we determine the values 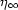 ≃ 0.9 and *N*_C_ ≃ 21 × 10^17^ photons mm^−2^. This indicates that the beam-induced damage in the polymer-containing structures is mediated by the polymer mold. A hypothesis for this effect is as follows. The Au gratings consist of segmented Au lines (see Fig. 1 ▶). Consequently, the breaking of the interface between a segmented Au line and the plating base only induces a local defect (Fig. 6 ▶). However, if the polymer mold is present, a local defect induces mechanical stress, which in turn may induce more defects (*cf.* Fig. 4). (iii) The Ir/Si gratings are found to be resistant to hard X-rays. We observe no degradation of the Ir/Si gratings upon exposing them to *N*_0_ ≃ 220 × 10^17^ photons mm^−2^ in air. We have verified this observation by SEM inspection of the Ir/Si grating after irradiation. We attribute this effect to the robust interface between Ir and Si, as obtained through atomic layer deposition of Ir (Vila-Comamala *et al.*, 2009 ▶). This result demonstrates the benefits of combining a low-*Z* material template with a high-*Z* material coating: template materials such as Si or diamond show a good thermal conductivity and low thermal deformations, while coating materials such as Ir provide a high diffraction efficiency. Table 1 ▶ summarizes the fit parameters.

## 4. Conclusions

Finally, we summarize the results of the present study in terms of guidelines for the planning and fabrication of future diffractive X-ray optics. (i) Organic compounds should be avoided in diffractive X-ray optics. If this is not possible, the use of a vacuum environment not only slows down the beam-induced damage but also reduces its effect on the diffraction efficiency. (ii) The effect of radiation damage on polymer-containing optics can also be reduced by overplating the diffractive structure, thereby increasing its chemical inertness. (iii) A possible approach to minimize the effect of radiation damage on electroplated X-ray optics is to use free-standing segmented metal lines. (iv) As demonstrated by the Ir/Si gratings used in the present study, atomic layer deposition of material leads to more robust interfaces, and hence to superior mechanical stability of the diffractive optics.

## Figures and Tables

**Table 1:** Parameters from the fit of equation (2) to the data of Fig. 5 ▶

Grating	Atmosphere	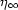	*N*_C_ (10^17^ mm^−2^)
pi/Au	Air	0.4	3.0 ± 0.5
pi/Au	Vacuum	0.7	13
Au	Air	0.9	21

## Footnotes

1 For a recent overview of X-ray microscopy, we refer to the proceedings of the 9th International Conference on X-ray Microscopy [*J. Phys. Conf. Ser.* (2009), Vol. 186].

2 We had to protect the detector from the high-power X-ray beam by adding attenuating filters in front of the detector. This led to beam hardening, * i.e.* we effectively filtered out the lower-energy X-rays from the spectral flux of the pink beam. Since the diffraction efficiencies of the gratings are strongly reduced for the higher-energy X-rays, we observed no diffraction pattern using the high-power X-ray beam.
